# Augmentation of lenvatinib efficacy by topical treatment of *miR-634* ointment in anaplastic thyroid cancer

**DOI:** 10.1016/j.bbrep.2021.101009

**Published:** 2021-05-09

**Authors:** Masahiro Kishikawa, Jun Inoue, Hidetoshi Hamamoto, Katsunori Kobayashi, Takahiro Asakage, Johji Inazawa

**Affiliations:** a Department of Molecular Cytogenetics, Medical Research Institute, Tokyo Medical and Dental University (TMDU), Tokyo, Japan; b Department of Head and Neck Surgery, TMDU, Tokyo, Japan; c MEDRx Co., Ltd., Kagawa, Japan; d Bioresource Research Center, TMDU, Tokyo, Japan

**Keywords:** miRNA therapeutics, Anaplastic thyroid cancer, Lenvatinib, Topical treatment, ATC, anaplastic thyroid cancer, TS-miR, tumor suppressive microRNA, *ASCT2*, alanine, serine, cysteine-preferring transporter 2

## Abstract

Anaplastic thyroid cancer (ATC) is one of the most lethal types of human tumors. Lenvatinib can improve the disease control and prognosis in patients with ATC. However, there is an unmet need to develop a therapeutically safer and non-invasive strategy that improves the efficacy of lenvatinib for advanced ATC tumors, which grow larger close to the skin. We previously demonstrated that the topical application of an ointment incorporating tumor suppressive microRNA (TS-miR), *miR-634*, is a useful strategy as a TS-miR therapeutics. Here, we found that the overexpression of *miR-634* synergistically increased lenvatinib-induced cytotoxicity by concurrently downregulating multiple genes related to cytoprotective processes, including *ASCT2*, a glutamine transporter, in ATC cell lines. Furthermore, the topical application of a *miR-634* ointment on subcutaneous tumors effectively augmented the anti-tumor effects of lenvatinib in an ATC xenograft mouse model. Thus, we propose topical treatment of a *miR-634* ointment as a rational strategy for improving lenvatinib-based therapy for ATC.

## Highlights

• Overexpression of *miR-634* induced apoptosis in ATC cell lines.
• Expression of *ASCT2* was upregulated by treatment with lenvatinib.
• *miR-634* increased lenvatinib-induced cytotoxicity by downregulating *ASCT2.*
• Topical application of a *miR-634* ointment enhanced efficacy of lenvatinib *in vivo.*
• Treatment with a *miR-634* ointment might be a rational strategy for ATC therapy.

## 1 Introduction

Anaplastic thyroid cancer (ATC) is the most aggressive human malignancy, accounting for approximately only 1% of all thyroid cancers [1,2]. It disproportionately contributes to thyroid cancer-related deaths because it is almost uniformly fatal [[2], [3], [4]]. Lenvatinib is an oral multi-tyrosine kinase inhibitor (TKI) that acts on vascular endothelial growth factor receptors 1–3 (VEGFR1-3), fibroblast growth factor receptors 1–4 (FGFR1-4), platelet-derived growth factor receptor-α (PDGFRα), and RET and KIT proto-oncogenes, and is involved in a marked improvement of disease control, as well as with radioiodine-refractory differentiated thyroid cancer [[5], [6], [7], [8], [9], [10]]. However, lenvatinib treatment results in a high incidence of adverse events (AEs), including hypertension, general fatigue and anorexia, protein urea, and tumor-skin fistula [[11], [12], [13]], therefore, dose reduction or treatment interruption are required until AEs resolve [12,14]. As such, there is an unmet medical need to develop a therapeutic strategy that improves the efficacy of lenvatinib.

MicroRNAs (miRNAs) can directly bind to the 3′ untranslated region (3′-UTR) in the multiple target transcripts and can downregulate the expression of those gene [15,16]. Tumor-suppressive miRNAs (TS-miRs) can directly target multiple genes related with oncogenesis and have the potential as an agent of anticancer drug [[17], [18], [19], [20]]. Our previous studies have demonstrated that *miR-634* overexpression effectively induced cell death by downregulating genes related with cytoprotective processes, including glutaminolysis, autophagy, antioxidant scavenging, anti-apoptotic signaling, mitochondrial homeostasis [19,20]. Furthermore, we demonstrated that a systemic administration of lipid nanoparticles (LNPs) including a synthetic double-strand (ds) *miR-634* mimic was therapeutically effective in xenograft mouse model [20]. Thus, the ds-*miR-634* mimic is a useful agent for cancer therapy.

The ionic liquid transdermal system (ILTS) has been used to enhance the transdermal permeability of nucleotides, including oligonucleotides and short interfering RNAs (siRNAs), *via* hydrophobic skin tissue to achieve efficient delivery into skin cells [21,22]. We recently developed an ointment containing ds-*miR-*634 mimics, *miR-634* ointment, using the ILTS, and demonstrated that the topical application of *miR-634* ointment suppressed tumor growth in a cutaneous squamous cell carcinoma (cSCC) xenograft mouse model and carcinogen-induced papilloma mouse model [23]. Importantly, *miR-634* overexpression synergistically enhanced epidermal growth factor receptor (EGFR) TKI-induced cytotoxicity through the reduction of glutaminolysis by downregulating *ASCT2*, a glutamine transporter in cSCC cells [23]. Thus, our previous reports suggested that overexpression of *miR-634* may increase the cytotoxicity induced by lenvatinib as a multi-TKI in ATC cells and topical application of *miR-634* ointment may be a non-invasive strategy for advanced ATC tumors, which grow larger close to the skin. Hence, in this study, we evaluated whether the topical application of *miR-634* ointment is useful strategy for improving the lenvatinib efficacy for ATC.

## 2 Material and methods

### 2.1 Cell culture

The cultures were maintained at 37 °C with 5% CO_2_ as described in previous papers [20,23]. Two ATC cell lines, 8505c and KTA-4, were obtained from JCRB (Japanese Collection of Research Bioresources) and were gifted by Dr. Akira Yoshida [24,25], respectively. 8505c cells were cultured in DMEM and KTA-4 cells were cultured in RPMI-1640 medium containing 10% fetal bovine serum (FBS). Once resuscitated, the cell lines were authenticated by monitoring cell morphology.

### 2.2 Antibodies and reagents

Antibodies against the following proteins were used: cleaved caspase-3 (#9661), cleaved PARP (#9541), and XIAP (#2042) (Cell Signaling Technology); β-actin (A5441), and TFAM (SAB1401383) (Sigma); LAMP2 (ab18529), OPA1 (ab42364), and APIP (ab98153) (Abcam); NRF2 (sc-13032) (Santa Cruz Biotechnology); and ASCT2 (Proteintech). Lenvatinib was purchased from Shelleckchem.

### 2.3 miRNA and siRNA synthesis and transfection

Transfection with miRNA or siRNA was performed using Lipofectamine RNAiMAX (Invitrogen) according to the manufacturer's instructions as described in previous papers [20,23]. The *miRVana miR-634* mimic and negative control 1 (*miR-NC*) were obtained from Thermo Scientific. The siRNA targeting *ASCT2* (M-007429-01) and negative control (*siNC*; D-001206-14) were obtained from Dharmacon Inc.

### 2.4 Assessment of the apoptotic cell population

As described in previous papers [20,23], apoptotic cells were stained with the MEBCYTO Apoptosis Kit (MBL), and cell population analysis was performed using an Accuri Flow Cytometer.

### 2.5 Cell survival assay

Cell survival was assessed by crystal violet (CV) staining as described in previous papers [20,23]. The optical density (OD) was measured at 560 nm using a microplate reader (SYNERGT H1) The percentage absorbance in each well was measured. The OD values of cells in control wells were arbitrarily set at 100% to calculate the percentage of viable cells.

### 2.6 Combination index (CI)

The CI was calculated using CalcuSyn (Biosoft) according to the methods reported by Chou and Talley as described in previous papers [20,23]. CI < 1 indicates a synergistic drug-drug interaction.

### 2.7 Immunofluorescence analysis

As described in previous papers [20], intracellular mitochondria were stained with 100 nmol/L MitoTracker Red CMX ROS (Life Technologies, Carlsbad, CA) for 30 min at 37 °C. After fixation with 10% trichloroacetic acid (TCA), images were obtained by confocal fluorescence microscopy (Nikon).

### 2.8 Measurement of ATP level

The levels of intracellular ATP per cells were measured by using Luminescent ATP Detection Assay Kit (Abcam) according to the manufacturers’ instructions as described in previous papers [23].

### 2.9 *In vivo* tumor growth assay

Animal experiments were carried out according to the guidelines and approval by the Tokyo Medical and Dental University Animal Care and Use Committee as described in previous papers [20,23]. Six-week-old female BALB/c nude mice were purchased from Charles River Laboratories. 8505c cells (1 × 10^7^ cells/100 μl in PBS) were subcutaneously injected into the right flanks of the mice (one injection per mice). On day 7 after tumor cell inoculation, miRNA ointment (*miR-NC* or *miR-634*; 10–20 μl/tumor) to subcutaneous tumors was topically applied. Lenvatinib was dissolved in 0.5% methylcellulose. Diluted lenvatinib (10 mg/kg) or vehicle (0.5% methylcellulose) were orally administrated three times a week. The tumor volume was calculated using the following formula: 4/3 × π × (shortest diameter × 0.5)^2^ × (longest diameter × 0.5).

### 2.10 Formulation of miRNA ointments

The miRNA ointment was formulated as described in previous papers [20,23]. The ILTS® (MEDRx) was used to formulate the ointments incorporating miRNAs. With this approach, ionic liquid is prepared from organic acids and amines. The molecular assembly involves the equilibrium reaction of ionic liquid/acid/amines and hydrogen bond interactions to improve the transdermal permeability of drugs or nucleotides in the hydrophobic field of skin tissue. The 0.2% ointment incorporating ds-*miR-NC* mimic or ds-*miR-634* mimic was formulated (2 mg miRNA/ml ointment).

### 2.11 *In situ* hybridization (ISH) analysis

The ISH analysis was performed using formalin-fixed, paraffin-embedded (FFPE) tissue sections according to the manufacturer's instructions (miRCURY LNA microRNA ISH Optimization Kit; Exiqon) as described in previous papers [20,23]. In brief, the sections were deparaffinized in xylene, rehydrated with a graded ethanol series, and incubated with Proteinase K for 10 min at 37 °C. Then, the sections were hybridized with digoxigenin (DIG)-labelled *miR-634* probes for 1 h at 55 °C, washed stringently, incubated with blocking agent for 15 min, and probed with a specific anti-DIG antibody (Sigma) directly conjugated to alkaline phosphatase (AP; Roche). AP converts the soluble substrates 4-nitro-blue tetrazolium (NBT) and 5-bromo-4-chloro-indolyl phosphate (BCIP) into a dark blue water- and alcohol-insoluble NBT-BCIP precipitate. Lastly, the sections were counterstained with nuclear fast red (Vector Laboratories).

### 2.12 Immunohistochemistry (IHC) analysis

The IHC analysis was performed as described in previous papers [20,23]. Nonspecific binding was blocked by incubation with goat serum in PBS. The slides were incubated overnight at room temperature with antibodies and the bound antibody was visualized with diaminobenzidine (Vector Laboratories), and the sections were lightly counterstained with hematoxylin.

### 2.13 qRT-PCR

As described in previous papers [20,23], Real-time qRT-PCR was performed using an ABI PRISM 7500 Fast Real-time PCR System according to the manufacturer's instructions. Gene expression values are presented as the ratio (difference in threshold cycle [Ct] values) between *miR-634* and an internal reference, *RNU6B*.

### 2.14 Western blotting

Western blotting was performed as described in previous papers [20,23]. After blocking with TBS containing 0.05% Tween 20 (Sigma) and 5% nonfat dry milk for 1 h, the membrane was incubated overnight with primary antibodies, washed and incubated for 1 h with horseradish peroxidase (HRP)-conjugated anti-mouse or anti-rabbit immunoglobulin G (IgG) secondary antibody, and were visualized using a LAS3000 imaging system (FUJIFILM).

### 2.15 Statistical analysis

Significance was assessed by the two‐tailed Student's *t*‐test or ANOVA (for multiple comparisons) using Prism version 5.04 (GraphPad) as described in previous papers [23]. Results with *p* ≤ 0.05 were considered statistically significant.

## 3 Results

### 3.1 Induction of apoptosis by overexpression of *miR-634* in ATC cells

**Fig. 1:**
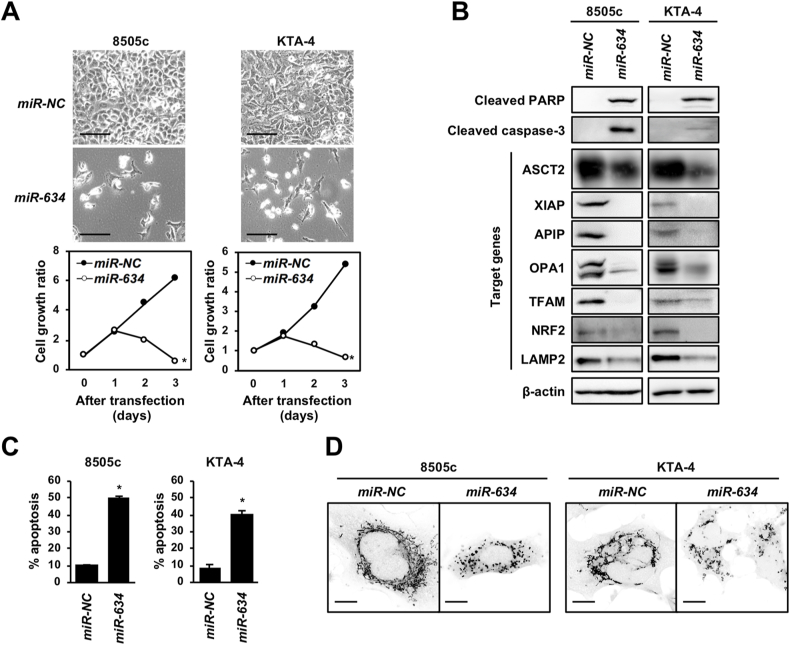
Induction of apoptosis by *miR-634* overexpression in ATC cells **A.** Phase-contrast images and growth rate of *miR-634*-transfected cells. Bar, SD of triplicate experiments. Scale bars; 50 μm. **B.** Western blotting analysis of *miR-634*-transfected cells. **C.** FACS analysis of the apoptotic cell population. Bar, SD of triplicate experiments. **D.** Representative images of mitochondrial staining. Scale bars; 2 μm. *P*-values were calculated using the two-sided Student's t-test (*P < 0.0001).

We have examined the effects of *miR-634* overexpression on cell survival of cell lines of multiple cancer types, including ATC [20]. We confirmed that *miR-634* overexpression effectively inhibited cell growth in ATC cell lines, 8505c and KTA-4 cells (Fig. 1A). In western blotting, the expression of the cleaved forms of caspase-3 and poly (ADP-ribose) polymerase (PARP) were markedly increased in *miR-634*-expressing cells (Fig. 1B). Furthermore, the expression levels of known *miR-634* target genes, including *ASCT2*, *XIAP*, *APIP*, *OPA1*, *TFAM*, *NRF2*, and *LAMP2*, were substantially reduced in *miR-634*-expressing cells, as expected (Fig. 1B). Fluorescence-activated cell sorting (FACS) analysis demonstrated that the apoptotic population as the annexin V and propidium iodide double-positive fraction was increased in *miR-634*-expressing ATC cells compared with that in *miR-NC*-expressing ATC cells as previously reported in other cancer cell lines [[19], [20], [23]] (Fig. 1C). A mitochondrial injury indicated by the fragmented morphology was observed in *miR-634*-expressing cells (Fig. 1D). These results indicated that overexpression of *miR-634* effectively induced apoptosis in ATC cells, as previously reported in other cancer cell lines [[19], [20], [23]], suggesting that the ds-*miR-634* mimic is a useful agent for TS-miR therapy in ATC.

### 3.2 Synergistic effects by combined treatment with *miR-634* and lenvatinib in ATC cells *in vitro*

**Fig. 2:**
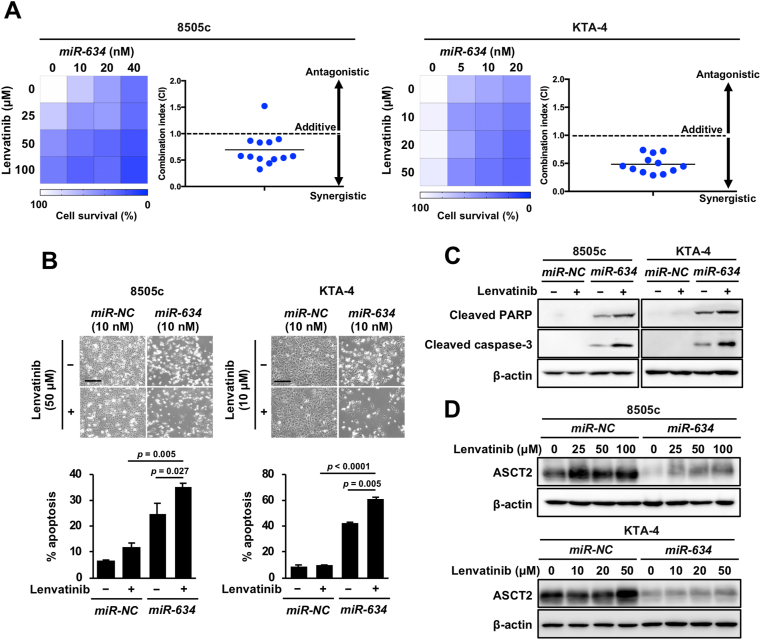
Synergistic effects by combined treatment with *miR-634* and lenvatinib in ATC cells *in vitro* **A.** The results are reported as the relative rate (%) in a 4 × 4 matrix experiment (mean of triplicate values) for combinations of *miR-634* and lenvatinib compared with non-transfected and non-treated cells. Respective concentrations are listed on x and y axes. The viability is color coded (white 100%, blue 0%). The combination index (CI) for each concentration were plotted as each graph for 8505c cells and KTA-4 cells. **B.** Increase of the apoptotic cell population. Scale bars; 50 μm. Bar, SD. *P*-values were calculated using two-way ANOVA. **C and D.** Western blotting analysis of apoptosis markers and ASCT2. In C, lenvatinib was treated with 50 μM in 8505c cells or 10 μM in KTA-4 cells, respectively, and *miR-NC* or *miR-634* were transfected with 10 nM. (For interpretation of the references to color in this figure legend, the reader is referred to the Web version of this article.)

Lenvatinib can improve the disease control and prognosis of ATC [12,13]. However, to minimize AE-associated dose reduction and treatment interruption, developing therapeutic strategy for the improvement of lenvatinib efficacy is required [14]. Thus, we evaluated whether *miR-634* overexpression enhanced lenvatinib efficacy in ATC cells *in vitro*. *miR-634* was transfected with increasing doses (10–40 nM for 8505c cells and 5–20 nM for KTA-4 cells) and the day after transfection lenvatinib (25–100 μM for 8505c cells and 10–50 μM for KTA-4 cells). The survival rate was decreased by combined treatment with lenvatinib and *miR-634* at varying doses. The combination index revealed a synergistic effect by *miR-634* and lenvatinib for both cell lines (Fig. 2A). Furthermore, FACS analysis revealed that the apoptosis frequency was further increased in cells treated with lenvatinib and *miR-634* compared with that in those with a single treatment (Fig. 2B). In western blotting, the expression of the cleaved forms of caspase-3 and PARP were increased in cells treated with the combined treatment (Fig. 2C). Thus, *miR-634* overexpression synergistically increased lenvatinib-induced cytotoxicity in ATC. Furthermore, we found that expression of ASCT2, a glutamine transporter, was upregulated by treatment with lenvatinib, and its increase was clearly reduced by *miR-634* overexpression in 8505c and KTA-4 cells (Fig. 2D). Also, the upregulation of ASCT2 expression following treatment with lenvatinib was shown in other ATC cell lines (Fig. S1). In addition, the lenvatinib-induced apoptotic cell death was markedly increased by siRNA-mediated inhibition of *ASCT2* (Fig. S2). Furthermore, we showed that the production of intracellular ATP as an energy source was markedly decreased in cells treated with lenvatinib and *miR-634* compared with that in those with a single treatment (Fig. S3). Taken together, these results suggest that overexpression of *miR-634* can enhance the efficacy of lenvatinib by triggering the energetic stress and the *miR-634*-mediated inhibition of *ASCT2* is partially involved in the enhancement of lenvatinib efficacy.

### 3.3 Augmentation of lenvatinib efficacy by topical application of *miR-634* ointment *in vivo*

**Fig. 3:**
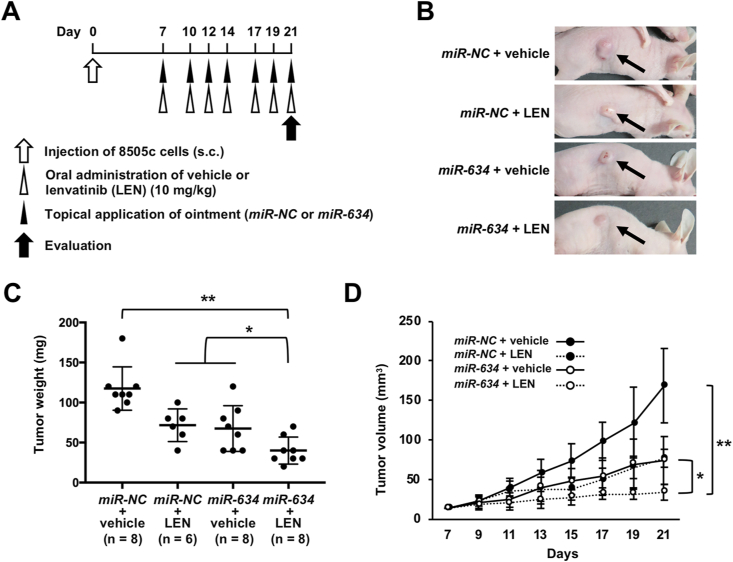
Augmentation of the efficacy of lenvatinib by topical treatment with *miR-634* ointment in the 8505c xenograft mouse model **A.** Experimental schedule for the application of *miR-634* ointment and treatment with lenvatinib. **B.** Representative images of resected tumors at day 21. **C and D.** Tumor weight (C) and tumor volume (D) in mice treated with *miR-NC* ointment + vehicle (n = 8), *miR-NC* ointment + lenvatinib (LEN) (n = 6), *miR-634* ointment + vehicle (n = 8), or *miR-634* ointment + lenvatinib (LEN) (n = 8). Bars; SD. Data are presented as the mean ± SD. *P*-values were calculated using two-way ANOVA (*P < 0.05, **P < 0.0001).

We recently formulated a *miR-634* ointment and demonstrated its therapeutic potential for cutaneous squamous cell carcinoma (cSCC) [23]. As the tumors are close to the skin in advanced ATC, *miR-634* ointment may be accessibly easy to apply on ATC tumors without medical devices. Thus, we examined whether topical treatment with the *miR-634* ointment improved the efficacy of lenvatinib in 8505c xenograft mice. Subcutaneous 8505c xenograft tumors was topically applied with *miR-NC* ointment or *miR-634* ointment, respectively, and mice were simultaneously administered the lenvatinib (10 mg/kg) or vehicle on days 7, 10, 12, 14, 17, 19, and 21 after cell injection (Fig. 3A). The tumors were resected 6 h after the final treatment on day 21. Tumor growth was more effectively inhibited by combined treatment with lenvatinib and *miR-634* ointment, compared in mice treated with lenvatinib and *miR-NC* ointment or vehicle and *miR-634* ointment (Fig. 3B–D).

**Fig. 4:**
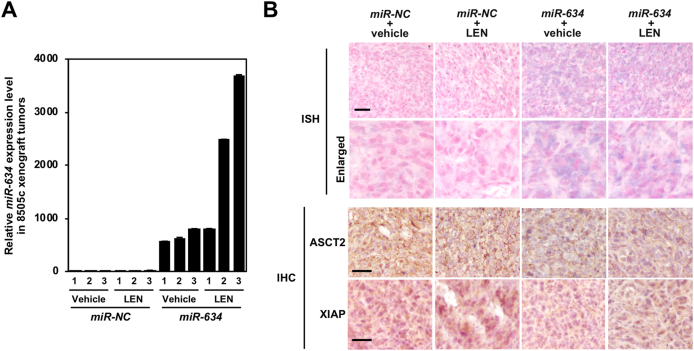
Delivery of *miR-634* and downregulation of the expression of *miR-634* target genes **A.** Expression analysis of *miR-634* in resected tumors by qRT-PCR. Bars; SD. Data are presented as the mean ± SD. **B.** ISH analysis of *miR-634* and IHC analysis in resected tumors. The *miR-634*-specific probe appears purple in the cytoplasm and the nucleus was counterstained with nuclear fast red. Scale bars; 50 μm.

Furthermore, we showed a marked increase of *miR-634* expression levels in tumors applied with *miR-634* ointment compared with tumors applied with *miR-NC* ointment by quantitative reverse transcriptase polymerase chain reaction (qRT-PCR) (Fig. 4A). *In situ* hybridization (ISH) analysis indicated the forced expression of *miR-634* as shown in purple stains in tumors applied with *miR-634* ointment (Fig. 4B). These observations suggest the effective delivery of *miR-634* into tumor cells. Moreover, based on immunohistochemical analysis, the expression of *miR-634* target genes, including ASCT2 and XIAP, decreased in tumors applied with *miR-634* ointment compared with tumors applied with *miR-NC* ointment (Fig. 4B). There was no change in body weight after any treatment (Fig. S4). Taken together, these results strongly suggest that topical application of *miR-634* ointment is reasonable as a therapeutic strategy to improve lenvatinib efficacy in ATC by concurrently modulating multiple cytoprotective processes.

## 4 Discussion

Lenvatinib, a multi-TKI taken orally once daily, is a breakthrough treatment for ATC, which previously had no effective treatment, however it is also involved in a high frequency of treatment-related AEs. The biggest problem in ATC treatment is that most patients with advanced ATC tumors develop tumor-skin fistula during treatment with lenvatinib. In this setting, dose interruptions are needed for the prevention of its spread [26]. Previous reports suggested that lenvatinib causes treatment-related carotid blowout syndrome, a life-threatening complication of head and neck cancer, leading to death in patients with invasion to the carotid artery [27,28]. To safely manage such severe treatment-related AEs in ATC patients, it is required to consider how to reduce the dose of lenvatinib while paying attention to rapid disease progression after discontinuing TKIs, known as flare phenomenon. Although manageable toxicities by dose adjustments of lenvatinib in patients with ATC were demonstrated in a previous study [29], the reduced dosage regimen remains controversial. In this study, we demonstrated that topical treatment with the *miR-634* ointment on subcutaneous tumors improved the lenvatinib efficacy in a human ATC xenograft mouse model, suggesting the therapeutic potential of the *miR-634* ointment combined with lenvatinib in human ATC therapy. Thus, this therapeutic strategy may be rational to improve the efficacy of lenvatinib to minimize the need for discontinued treatment and maximize patient outcomes by improving the quality of life [14]. Furthermore, advanced ATC tumors grown larger close to the skin and tumor-skin fistulas develop during lenvatinib treatment. As *miR-634* ointment can be easily applied to tumors without medical devices and can be self-administrated together with lenvatinib at home, it may be advantageous in terms of convenience and ease of use for patients with advanced ATC.

To supply the bioenergetic and biosynthetic demands for cell survival, metabolic pathways, including glutaminolysis, are utilized in cancer cells [23,30]. It has been known that glycolysis is suppressed by treatment with TKIs [23,31,32]. In addition, the suppression of glutaminolysis enhances the TKI efficacy through the induction of energetic stress, suggesting that dual suppression of glycolysis and glutaminolysis is reasonable as the therapeutic strategy for cancer [23,33,34]. Our previous findings have demonstrated that treatment with EGFR-TKIs, such as gefitinib and erlotinib, upregulated ASCT2 expression, and its inhibition of *ASCT2* through *miR-634* overexpression suppressed glutaminolysis and augmented TKI-induced cytotoxicities *via* triggering severe energetic stress in A431 cells [23]. Similarly, in the present study, the expression level of ASCT2 was markedly upregulated by treatment with lenvatinib and its knockdown increased lenvatinib-induced cytotoxicity in 8505c cells, suggesting that the inhibition of *ASCT2* by *miR-634* overexpression is partially involved in the enhancement of lenvatinib efficacy. However, clarification of the metabolic significance of lenvatinib-induced upregulation of ASCT2 expression and the status of energetic stress induced by the combined treatment will be needed to understand the synergistic mechanism for combined treatment with *miR-634* and lenvatinib.

It has been demonstrated that the ILTS is useful for the transdermal permeability of nucleotides in skin cells [23,35,36]. Using the ILTS, we showed that *miR-634* was efficiently delivered into tumor cells in ATC xenograft mice and downregulated the expression of target genes. Moreover, this was also recently observed in a cSCC xenograft mouse model and DMBA/TPA-induced papilloma mouse model [23]. In general, it has been known that RNAs are not stable for the degradation by nucleases in the skin and blood circulation [23,37]. Hence, the chemical modification for the synthesis of ds-*miR-*634 mimics may contribute to further enhance the delivery of *miR-634* into tumor cells [23,38]. Thus, the optimization of the *miR-634* ointment is necessary at the preclinical stage. In addition, the safety of the *miR-634* ointment, including the therapeutic schedule and dose, needs to be further examined in a large cohort of mice for the clinical use of miR therapeutic option.

## Declaration of competing interest

The authors declare the following financial interests/personal relationships which may be considered as potential competing interests:

J.I. received a research grant from Otsuka Pharmaceutical Co., Ltd.
