# Supplementary material for: Augmentation of lenvatinib efficacy by topical treatment of miR-634 ointment in anaplastic thyroid cancer
Source: Biochem Biophys Rep. 2021 May 9;26:101009. doi: 10.1016/j.bbrep.2021.101009 (PMC8131394; doi:10.1016/j.bbrep.2021.101009)
Supplement: Multimedia component 1 [file mmc1.docx]

**Supplementary Figure legends**

**Figure S1**

**Western blotting analysis of ASCT2 in ATC cells.**

Cell lysates were separated by SDS-PAGE and immunoreacted with the indicated antibodies.

**Figure S2**

**Augmentation of lenvatinib-induced cytotoxicity by *ASCT2* knockdown**

**A.** Western blotting analysis of ASCT2. Cell lysates were separated by SDS-PAGE and immunoreacted with the indicated antibodies.

**B.** Cell survival assay. 8505c were transfected with 10 nM *NC* siRNA (*siNC*) or *ASCT2* siRNA (*siASCT2*) and simultaneously treated with 100 µM of lenvatinib. After 24 h of lenvatinib treatment, the apoptotic population was measured by FACS analysis. *P*-values were calculated using two-way ANOVA.

**Figure S3**

**The level of intracellular ATP**

Intracellular ATP levels were measured and normalized to the cell survival rate. Error bars indicate the SD. Relative levels are presented as mean ± SD. *P*-values were calculated using the two-sided Student's t-test.

**Figure S4**

**Measurement of mouse body weight at 7 (pre-treatment) and 21 (post-treatment) days after the injection of 8505c cells.**

Data are presented as the mean ± SD. Bars; SD. *P*-values were calculated using the two-sided Student's t-test. *P* = 0.70 in mice treated with *miR-NC* ointment + vehicle (n = 8), *P* = 0.86 in *miR-NC* ointment + lenvatinib (LEN) (n = 6), *P* = 0.96 in *miR-634* ointment + vehicle (n = 8), or *P* = 0.82 in *miR-634* ointment + lenvatinib (LEN) (n = 8). NS; not significance.
