# Supplementary figures and images for: Augmentation of lenvatinib efficacy by topical treatment of miR-634 ointment in anaplastic thyroid cancer
Source: Biochem Biophys Rep. 2021 May 9;26:101009. doi: 10.1016/j.bbrep.2021.101009 (PMC8131394; doi:10.1016/j.bbrep.2021.101009)

Figure S1

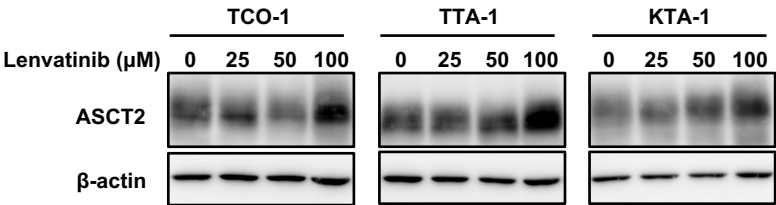

Figure S2

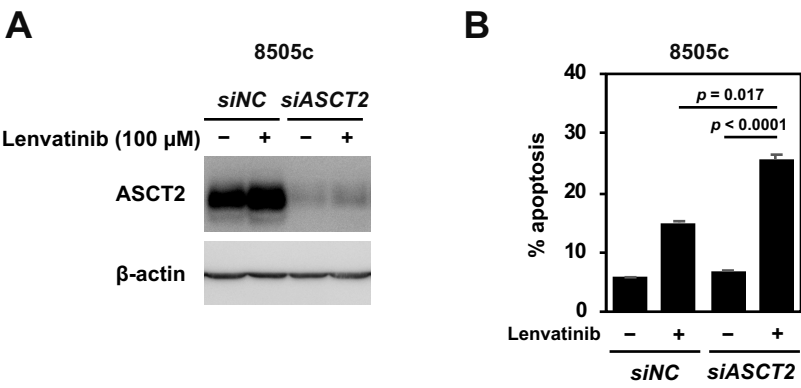

Figure S3

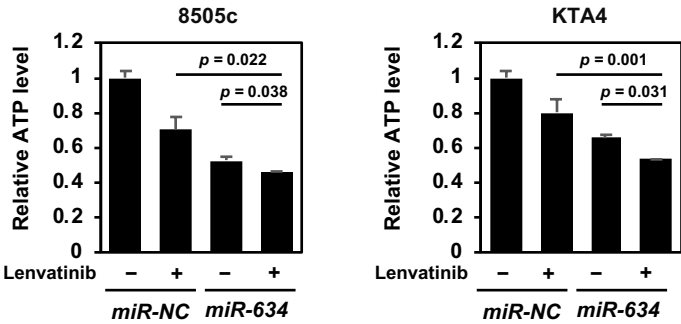

Figure S4

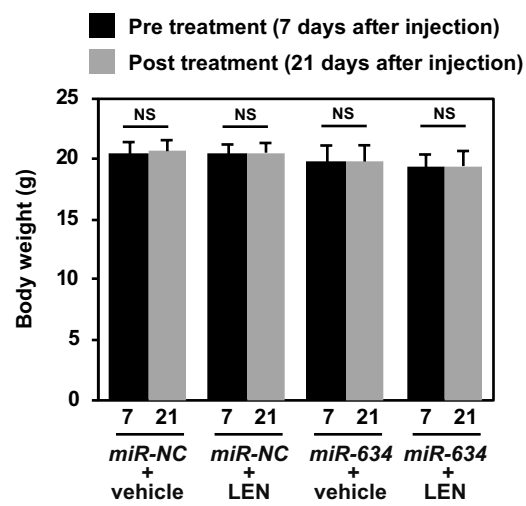

Supplement: Multimedia component 2 [file mmc2.pdf]
